# Reconsidering Placebo Effects in Neuromodulation for Parkinson’s Disease: Lessons for Clinical Trials and Therapeutic Translation

**DOI:** 10.3390/biomedicines14030532

**Published:** 2026-02-27

**Authors:** Aybike Reyhanli, Jorge Ortega-Márquez, Carla Pastora-Sesin, Joao Pedro Perin, Anna Carolyna Gianlorenço, Lucas Camargo, Felipe Fregni

**Affiliations:** 1 Department of Chemical Engineering, University of Massachusetts Lowell, Lowell, MA 01854, USA; aybike_reyhanli@uml.edu; 2 Neuromodulation Center and Center for Clinical Research Learning, Spaulding Rehabilitation Hospital and Massachusetts General Hospital, Harvard Medical School, Boston, MA 02115, USA; jortegamarquez@mgb.org (J.O.-M.); c.pastorasesin@gmail.com (C.P.-S.); jperin@mgb.org (J.P.P.); alepesteurgianlorenco@mgh.harvard.edu (A.C.G.); lcamargo@mgh.harvard.edu (L.C.); 3 Laboratory of Neuroscience and Neurological Rehabilitation, Physical Therapy Department, Federal University of São Carlos, São Carlos 13565-905, SP, Brazil

**Keywords:** placebo, Parkinson’s disease, neuromodulation

## Abstract

**Background**: Placebo effects are well documented in Parkinson’s disease (PD) clinical trials and represent a major methodological challenge in interpreting neuromodulation studies. Although sham stimulation has been associated with clinical improvement, the magnitude, durability, and outcome specificity of placebo-related effects across non-invasive neuromodulation trials remain incompletely characterized. **Methods**: This systematic review and meta-analysis followed PRISMA guidelines and was registered in PROSPERO (CRD1272381). PubMed/MEDLINE, Embase, Web of Science, and the Cochrane Library were searched from inception through September 2025. Randomized, sham-controlled trials of non-invasive neuromodulation in adults with PD were included. **Results**: Seventeen randomized sham-controlled trials (n = 654 participants) involving repetitive transcranial magnetic stimulation and transcranial direct current stimulation were included. Sham stimulation was associated with small but statistically significant improvements in UPDRS Part III (motor examination) at post-intervention and follow-up, whereas no significant placebo-related improvement was observed for UPDRS Total score. Placebo effects were modest and did not increase over time. In contrast, active neuromodulation produced larger and more durable improvements in both UPDRS Total and Part III, with statistically significant effects maintained at follow-up. **Conclusions**: Placebo effects contribute to short-term clinical improvement in non-invasive neuromodulation trials for PD, particularly for motor examination outcomes, but do not fully account for the sustained benefits observed with active stimulation. Placebo responsiveness is outcome- and time-dependent, underscoring the importance of rigorous trial design, including careful outcome selection, assessment timing, expectancy management, and comparator structures, to accurately estimate neuromodulation efficacy and support clinical translation.

## 1. Introduction

The placebo effect refers to a psychobiological phenomenon in which measurable clinical improvement occurs following the administration of a physically and pharmacologically inactive intervention [1,2,3]. Various external cues and internal processes, such as expectations, prior learning, and the therapeutic context, mediate the placebo effect [1]. Once regarded as a nonspecific artifact, placebo responses are now recognized as active processes capable of producing reproducible changes in symptom expression across a range of neurological and psychiatric conditions [4,5]. However, the magnitude of the placebo effect varies among patients depending on disease pathology, disease severity, treatment invasiveness, study protocol, and individual patient characteristics such as sex and age [6]. Consequently, interpreting clinical trial results in neurological and psychiatric disorders is particularly challenging, as this variability complicates isolating the actual therapeutic impact of the active interventions [7].

Parkinson’s disease (PD) represents a unique condition for studying placebo effects due to several factors. The dopamine release in the striatum along with reductions in firing rate and bursting activity in the subthalamic nucleus during sham interventions are closely linked to PD pathophysiology and result in improved motor function, particularly bradykinesia and rigidity [8,9]. These symptoms are often assessed in research and clinical practice using standardized clinician-rated scales, such as Movement Disorder Society-Sponsored Revision of the Unified Parkinson’s Disease Rating Scale (MDS-UPDRS) [10], which enables a quantitative evaluation of clinical outcomes [4]. For example, previous studies evaluating the placebo effect of medical and surgical interventions reported an overall improvement of 16% in motor symptoms, with a large range between 0 and 55% [11,12]. However, substantial heterogeneity in clinical outcomes and evaluation parameters persists across clinical trials.

PD symptoms are primarily managed by dopaminergic medications and surgical interventions [13]. However, the therapeutic effectiveness of medical treatment declines over time, and fewer than 5% of patients are eligible for surgical interventions [14]. Consequently, innovative treatments such as non-invasive neuromodulation techniques have been intensively investigated [15]. Understanding the limits of the placebo response in these techniques is crucial for interpreting the therapeutic effect in clinical trials [16]. Goetz et al. [12] demonstrated that PD patients receiving sham surgical interventions exhibited a placebo response more than four times larger than those with sham medical treatments, highlighting the variability of the placebo responsiveness and its potential to obscure true treatment effects. As with other intervention modalities, such as drugs and physical exercise, understanding placebo effect in neuromodulation is necessary to interpret treatment efficacy and to improve clinical trial design, particularly in the context of neurodegenerative conditions like PD [17]. Although numerous studies investigating non-invasive neuromodulation techniques for PD have emerged in the past two decades [18], the magnitude and consistency of placebo-related clinical improvement across neuromodulation trials remain incompletely synthesized. This gap complicates the interpretation of the true neuromodulation effects, particularly for outcomes reflecting both overall disease burden and motor impairment.

The objective of this systematic review and meta-analysis is to characterize the placebo effect of non-invasive neuromodulation techniques in PD using UPDRS. Our findings aim to provide essential insights into the placebo effect associated with neuromodulation techniques in PD. Given the increasing use of neuromodulation as an adjunct or alternative to pharmacologic therapy, clarifying the clinical impact of placebo effects will be helpful for accurate trial interpretation, optimization of sham-controlled clinical trial designs, and contextualization of therapeutic benefit.

## 2. Materials and Methods

This systematic review and meta-analysis were conducted following the “Preferred Reporting Items for Systematic Reviews and Meta-Analyzes” (PRISMA) Statement guidelines [19] and the Cochrane Handbook for Systematic Reviews of Interventions [20]. The protocol for this study was registered in the International Prospective Register of Systematic Reviews (PROSPERO) under the registration number CRD1272381.

PubMed/MEDLINE, Web of Science, Cochrane Library, and Embase databases were searched from inception until September 2025. A search strategy was developed using a combination of the following key terms as keywords: Parkinson’s disease (Parkinson’s disease, PD); Placebo/Sham (placebo, placebo effect, sham); Non-invasive Brain Stimulation (neuromodulation, transcranial magnetic stimulation, transcranial direct current stimulation, transcranial pulse stimulation, vagus nerve stimulation, static magnetic field stimulation); and standard clinical outcomes measurement (Unified Parkinson’s Disease Rating Scale, UPDRS). Full details about the search strategies are available in Supplementary Table S1.

The inclusion criteria for this study were as follows: randomized placebo-controlled trials (RCTs) involving adult patients diagnosed with PD; clinical outcomes by UPDRS, which is considered the current gold standard for measuring clinical outcomes for PD [21], any type of non-invasive brain stimulation; and studies available in full text and published in English or Spanish. Studies were excluded if they were preclinical or animal studies, observational designs, systematic reviews, meta-analyses, editorials, letters to the editor, conference abstracts, or other non-peer-reviewed literature. Studies without a sham or placebo comparison arm or those not reporting clinical outcomes and trials evaluating combined or multimodal interventions (e.g., neuromodulation in combination with physical therapy) were also excluded unless separate outcome data for the neuromodulation intervention were reported.

Eligibility assessment was performed following a flowchart sequence. Two independent reviewers (A.R. and P.P.) conducted study selection and data extraction. Disagreements or inconsistencies were addressed with a third independent reviewer (J.O.). When reported, the following variables were extracted from each study: author, year of publication, country, study design, population characteristics such as age, sex, time since diagnosis, details of the intervention and placebo non-invasive brain stimulation techniques (type of device, session duration and frequency, stimulation intensity), and available sections from the UPDRS at baseline, immediate post-intervention and first follow-up measurement. If UPDRS data were unavailable, outcome values were extracted from published graphs using WebPlotDigitizer (version 4.8; Automeris LLC, Austin, TX, USA), when possible [22]. If data from graphs were not available, authors were contacted via email. Studies with unresolved data gaps after the authors were contacted by email were excluded to avoid introducing bias through imputation or assumption-based methods.

Assessment of the quality of the studies was conducted using version 2 of the Cochrane Risk of Bias tool for randomized trials (RoB 2), which uses an algorithm to rate each domain as ‘low risk of bias,’ ‘some concerns,’ or ‘high risk of bias.’ The overall risk of bias reflects the highest level of concern across the five domains [20,23]. Two independent reviewers (A.R. and P.P.) evaluated each study’s quality, considering selection, detection, performance, attrition, and reporting biases. Discrepancies were solved with the support of a third author (J.O.). Publication bias assessment was not conducted due to the small number of included studies.

Statistical analyses were conducted using RStudio (Posit Software, PBC, Boston, MA, USA; build 2025.09.0+387.pro3) with the meta (version 8.2-1) and metafor (version 4.8-0) packages). Data are presented as mean ± standard deviation (SD) and in case only the standard error of the mean (SEM) was reported, it was converted to SD to standardize measures of variability, using the formula: SD = SEM × √n, where *n* represents the sample size [24]. Meta-analyses of active versus placebo interventions were conducted for available UPDRS outcomes at post-intervention and follow-up using a random-effects inverse-variance model, accounting for between-study heterogeneity [25]. Within-placebo group analyses were conducted using the metamean function with the restricted maximum likelihood (REML) estimator [26]. Effect sizes were calculated as the standardized mean difference (SMD) using Cohen’s d, and between-study heterogeneity was estimated using REML. As most included studies did not report pre–post correlation coefficients, standardized mean differences for within-group analyses were calculated using available summary statistics without adjustment for within-subject correlation, consistent with Cochrane guidance when such data are unavailable [27]. In addition, a conservative threshold of three studies per comparison was adopted to improve the stability and reliability of pooled estimates, as recommended in the Cochrane Handbook for Systematic Reviews of Interventions [27,28]. Effect sizes were interpreted as reflecting improvement or decline in UPDRS scores depending on the direction of the comparison: in within-placebo analyses, positive values indicated improvement following sham intervention, whereas in between-group analyses, positive values indicated greater improvement in the sham group compared with active intervention. Effect size magnitudes were classified as small (<0.5) moderate (0.5–7.9), and large (≥0.8) [29]. Statistical heterogeneity was assessed using the I^2^ statistic, with 25%, 50%, and 75% considered low, moderate, and high, respectively [30].

## 3. Results

### 3.1. Study Selection

**Figure 1:**
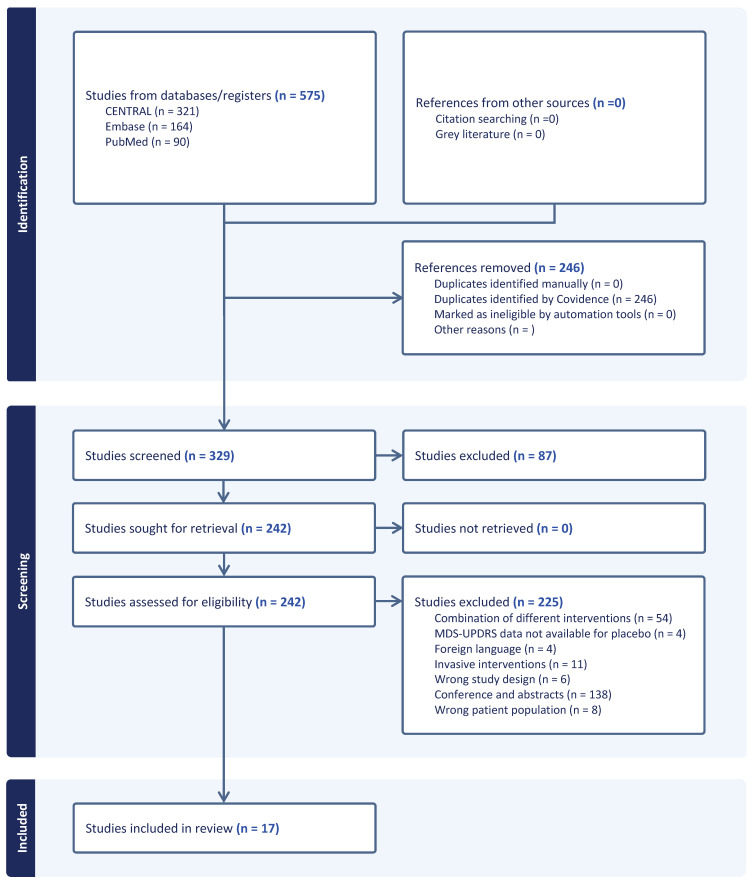
PRISMA flow diagram showing search results and literature selection.

The database search identified 575 records, including 321 from CENTRAL, 164 from Embase, and 90 from PubMed, with no additional records identified through citation searching or grey literature. After removal of 246 duplicate records, 329 studies were screened based on titles and abstracts, of which 87 were excluded. The remaining 242 full-text articles were assessed for eligibility. Of these, 225 studies were excluded for the following reasons: combination of different interventions (n = 54), conference or abstracts publications (n = 140), invasive interventions (n = 11), ineligible patient population (n = 8), ineligible study design (n = 6), UPDRS data not available for the placebo arm (n = 4), and publications in languages other than English or Spanish (n = 4). Ultimately, 17 studies met the inclusion criteria and were included (Figure 1).

### 3.2. Study Characteristics

A total of 654 individuals diagnosed with PD were enrolled across the included studies, with 285 participants assigned to sham intervention groups. All studies were reported as randomized, sham-controlled clinical trials. Despite the search strategy included several non-invasive neuromodulation modalities, only randomized sham-controlled trials using repetitive transcranial magnetic stimulation (rTMS) and transcranial direct current stimulation (tDCS) met the eligibility criteria and provided sufficient data for inclusion. Thirteen studies employed rTMS [31,32,33,34,35,36,37,38,39,40,41], while four made use of transcranial direct current stimulation (tDCS) [42,43,44,45]. Regarding the studies using rTMS, the primary motor cortex (M1) [32,33,34,35,38,41], supplementary motor area (SMA) [31,37], and dorsolateral prefrontal cortex (DLPFC) [32,33,34,39] were the most frequently targeted cortical regions. A subset of studies additionally stimulated the motor hand area [36], parietal cortex [40], and occipital cortex [38]. The stimulation frequencies ranged from 0.2 [38] to 50 Hz [34,35], with the number of pulses per session varying between 100 [38] and 4000 [32,33] per region. The duration of each session ranged from 17 [31] to 40 min [32,33], and the number of stimulation sessions ranged from one [36] to twenty [32,33]. These stimulation parameters and cortical targets are consistent with early randomized rTMS trials in PD, in which M1 and SMA were identified as the most promising targets for motor symptom modulation [45]. Of the studies using tDCS, three applied anodal stimulation [42,43,44], while one employed both anodal and cathodal stimulation [45]. The intensity of stimulation ranged from 1 to 2 milliamps (mA) [42,43,44,45], with each session lasting between 15 [44] and 20 min [42,43,45]. The total number of sessions ranged from one [44,45] to eight [43], consistent with established safety and dosing recommendations for tDCS in PD populations. All studies reported clinical outcomes using the UPDRS Total score and the motor examination component (UPDRS Part III). Follow-up assessments were generally short- to medium-term, ranging from 2 to 84 days across studies, with most trials reporting outcomes at approximately 2–4 weeks after the intervention. Supplementary Table S2 provides further methodological and stimulation details for each study.

### 3.3. Quality of the Studies

Overall, the included randomized controlled trials demonstrated low risk of bias in several key domains, particularly regarding deviations from intended interventions (D2) and outcome measurement (D4), which were consistently rated as low risk across most studies [32,33,34,35,37,38,39,41]. Some concerns were frequently identified, most commonly related to the randomization process (D1) and missing outcome data (D3), reflecting limited reporting of allocation procedures or incomplete outcome reporting in earlier trials [40,42,44,45,46,47]. In addition, bias in the selection of the reported result (D5) was judged as having some concerns in all studies, largely due to insufficient detail on prespecified analyses. Consequently, while no study was rated as having a high overall risk of bias, most trials were classified as having “some concerns” in the overall judgment, reflecting cumulative methodological limitations rather than critical flaws in individual domains. A detailed domain-level assessment is provided in Supplementary Figure S1.

### 3.4. Clinical Effects of rTMS on PD

#### 3.4.1. Placebo-Related Effects on UPDRS Outcomes

**Figure 2:**
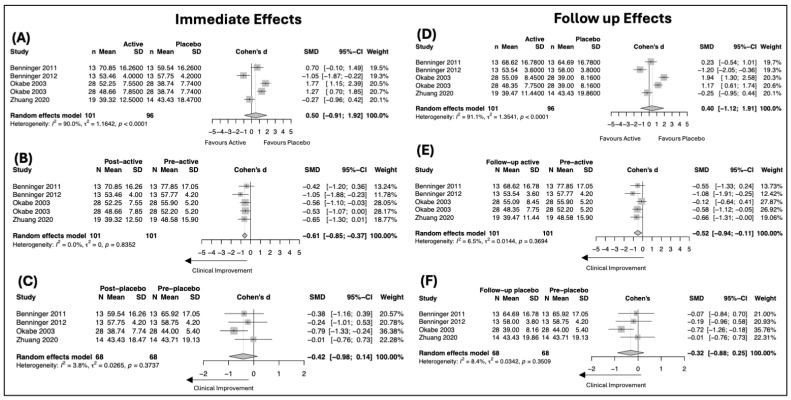
Forest plots showing standardized mean differences (SMDs) and 95% confidence intervals (CIs) for changes in the Unified Parkinson’s Disease Rating Scale (UPDRS) Total score. Panels depict between-group comparisons of active versus sham stimulation at (**A**) post-intervention and (**D**) follow-up, within-group changes following active stimulation at (**B**) post-intervention and (**E**) follow-up, and within-group changes following sham stimulation at (**C**) post-intervention and (**F**) follow-up. Negative SMD values indicate clinical improvement (reduction in the scores).

**Figure 3:**
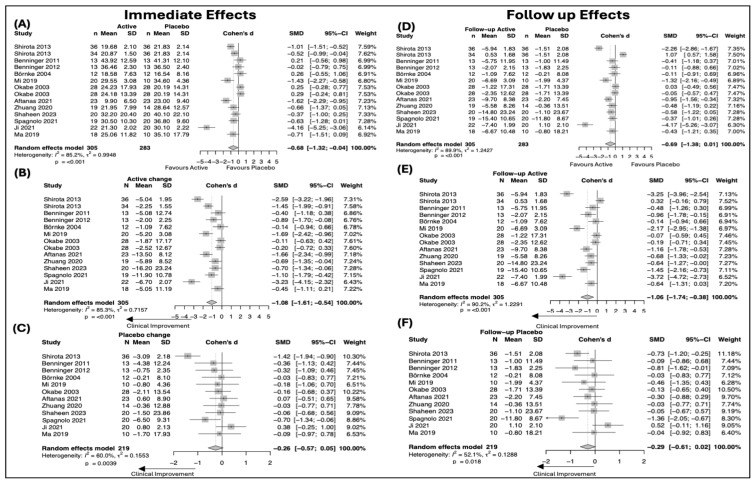
Forest plots showing standardized mean differences (SMDs) and 95% confidence intervals (CIs) for score changes in the UPDRS motor examination component (UPDRS Part III). Panels depict between-group comparisons of active versus sham stimulation at (**A**) post-intervention and (**D**) follow-up, within-group changes following active stimulation at (**B**) post-intervention and (**E**) follow-up, and within-group changes following sham stimulation at (**C**) post-intervention and (**F**) follow-up. Negative SMD values indicate clinical improvement (reduction) in the scores.

Across the included sham-controlled trials, placebo (sham) stimulation was associated with small and inconsistent changes in UPDRS outcomes (Figure 2 and Figure 3). For UPDRS Total score, within-placebo analyses did not demonstrate a statistically significant improvement either immediately after intervention (SMD = −0.42, 95% CI −0.98 to 0.14; I^2^ = 3.8%) or at follow-up (SMD = −0.32, 95% CI −0.88 to 0.25; I^2^ = 8.4%), indicating the absence of a robust global placebo effect on overall disease severity. In contrast, for UPDRS Part III (motor examination), placebo stimulation was associated with a small but statistically significant improvement at post-intervention (SMD = −0.26, 95% CI −0.57 to −0.05; I^2^ = 60%). This improvement remained at follow-up, though the magnitude remained small (SMD = −0.29, 95% CI −0.72 to 0.02; I^2^ = 52.1%). Overall, placebo effects were more evident for motor outcomes than for global disease measures, were small in magnitude, and showed no evidence of progressive strengthening over time.

#### 3.4.2. Active Intervention Effects on UPDRS Outcomes

Active neuromodulation was associated with clinically meaningful improvements in both global disease severity and motor symptoms, with effects that were maintained at follow-up (Figure 2 and Figure 3). For UPDRS Total score, within-group analyses demonstrated a significant improvement immediately after intervention (SMD = −0.61, 95% CI −0.85 to −0.37; I^2^ = 0%). Importantly, this improvement persisted at follow-up, remaining statistically significant despite a modest reduction in effect size (SMD = −0.52, 95% CI −0.94 to −0.11; I^2^ = 6.5%). For UPDRS Part III, active stimulation produced a large improvement at post-intervention (SMD = −1.08, 95% CI −1.61 to −0.54; I^2^ = 85.3%). This effect was sustained at follow-up, with a comparable magnitude of benefit (SMD = −1.06, 95% CI −1.74 to −0.38; I^2^ = 90.2%). Overall, active intervention effects were robust and durable, particularly for motor outcomes, with significant improvements maintained beyond the immediate post-intervention period.

Between-group comparisons of active versus sham stimulation did not demonstrate a statistically significant difference for UPDRS Total score at post-intervention (SMD = 0.50, 95% CI −0.91 to 1.92; I^2^ = 90.0%) or at follow-up (SMD = 0.40, 95% CI −1.12 to 1.91; I^2^ = 91.1%). For UPDRS Part III, active stimulation was superior to sham at post-intervention (SMD = −0.68, 95% CI −1.32 to −0.04; I^2^ = 85.2%), whereas this between-group difference was no longer statistically significant at follow-up (SMD = −0.69, 95% CI −1.38 to 0.01; I^2^ = 89.9%).

## 4. Discussion

This review evaluated placebo and active effects of non-invasive neuromodulation in Parkinson’s disease using standardized global and motor clinical outcomes across sham-controlled trials. Both active and placebo interventions were associated with short-term clinical improvement, indicating a measurable placebo component in neuromodulation trials. However, active stimulation demonstrated more consistent and durable effects, particularly for motor outcomes, while placebo-related improvements were smaller, outcome-dependent, and attenuated over time. These findings suggest that placebo effects contribute to immediate clinical change but do not fully account for the sustained therapeutic signal observed with active neuromodulation.

### 4.1. Placebo Effects Are Outcome-Specific and Limited in Magnitude

Our findings demonstrate that placebo effects in neuromodulation trials for Parkinson’s disease are not uniform across clinical outcomes. Sham stimulation was associated with small but statistically significant improvements in UPDRS Part III at both post-intervention and follow-up, whereas no significant placebo-related change was observed for UPDRS Total score. This divergence indicates that motor examination outcomes may be more susceptible to placebo-related influences than composite measures of global disease severity. Similar outcome-specific placebo effects have been reported across neuromodulation trials in other neurological and psychiatric conditions, where sham stimulation produces measurable but modest symptom changes depending on the outcome domain assessed [48,49,50,51].

In a large systematic review published in the *New England Journal of Medicine* [52], the authors critically examined the magnitude of placebo effects by comparing placebo-treated groups with no-treatment controls across a broad range of randomized clinical trials. Their analysis demonstrated that placebo interventions had little to no effect on objective or hard clinical outcomes, whereas small to moderate effects were observed for subjective or observer-dependent measures, particularly those related to pain and self-reported symptoms. The authors concluded that placebo effects are not universal but are highly dependent on outcome type, with greater apparent effects in domains susceptible to reporting bias, expectation, and contextual influences. Although UPDRS Total is also observer-dependent, it likely showed minimal placebo responsiveness because participants may not have expected meaningful short-term changes in global scores. In contrast, motor performance (UPDRS Part III) is more immediately observable and may be more susceptible to expectancy-driven effects.

UPDRS Part III measures observable motor performance at a single clinical examination and is sensitive to short-term fluctuations in motor state, motivation, and expectancy. In contrast, UPDRS Total aggregates multiple domains, including non-motor and functional components, which may dilute transient expectancy-related effects. These domains reflect broader functional and non-motor aspects of disease severity, which are typically less responsive to short-term contextual influences than the motor examination in Part III [10,53]. This distinction is consistent with prior placebo analyses in non-invasive brain stimulation trials, suggesting that domain-specific outcomes are more likely to capture short-term placebo responsiveness, whereas composite or multidomain measures provide a more conservative estimate of clinical change [45,49,50,51]. The absence of a significant placebo effect on UPDRS Total in our analysis therefore supports the interpretation that global disease severity is less responsive to contextual or expectancy-driven mechanisms than motor performance alone.

The observed pattern aligns with prior mechanistic work in Parkinson’s disease indicating that placebo responses are primarily driven by expectancy-related processes. Placebo responses in PD are associated with rapid, transient dopaminergic release in the striatum, leading to immediate subjective and occasionally observable motor changes without durable physiological modification [8,16]. Experimental and clinical studies across neuromodulation trials further suggest that placebo-induced dopaminergic activation occurs within minutes and predominantly affects short-term perception and performance rather than sustained motor function [48,50,51]. In contrast, active neuromodulatory interventions are thought to induce longer-lasting plastic changes at network and circuit levels, which persist beyond the acute expectancy window and are reflected in maintained clinical effects at follow-up [16,51].

To further contextualize the outcome-specific effects and their clinical relevance, prior research suggests a minimal clinically important difference (MCID) of approximately 3–3.5 points for UPDRS Part III and about 4–10 points for the UPDRS Total score [54,55]. The placebo-related effects observed in our analysis are therefore likely below thresholds for clearly perceptible clinical improvement. Importantly, placebo-related improvements in the present analysis remained small in magnitude (SMDs approximately −0.3 to −0.4) and did not demonstrate progressive strengthening over time, indicating that sham stimulation does not produce cumulative or durable clinical change, consistent with prior systematic reviews using sham neuromodulation in different medical conditions [49,50,51].

### 4.2. Implications for the Design of Clinical Trials

The present findings underscore the importance of carefully addressing expectancy-related effects in sham-controlled neuromodulation trials for Parkinson’s disease. Although placebo-related improvements were modest, their preferential effect in motor examination outcomes highlights how trial context and outcome selection can influence observed effects because participants may anticipate motor improvement in these trials. Future studies should therefore aim to minimize unintentional expectancy amplification through standardized, neutral communication with participants. For example, prior neuromodulation trials have varied considerably in how blinding strategies are implemented, with most of the studies emphasizing the potential benefits in motor symptoms. Such framing may differentially affect outcomes like UPDRS Part III, which are sensitive to short-term performance and motivation. Using harmonized scripts that emphasize uncertainty and equipoise, as well as avoiding symptom-specific promises, may help reduce variability attributable to expectancy [56,57]

In addition to managing expectations, measuring expectancy directly represents an important methodological advance. A narrative review in non-invasive brain stimulation modalities observed that subjects are prone to placebo effect due to their expectation and beliefs on the treatment provided. Incorporating brief expectancy or credibility questionnaires at baseline—and potentially during treatment—would allow expectancy to be examined as a prespecified moderator rather than an unmeasured confound. Previous evidence demonstrates expectation in PD enhances the release of dopamine in the striatum, particularly when participants believe they will receive the active treatment (e.g., 75% probability), but not at lower expectations [58]. This approach may be particularly relevant in PD, where placebo responsiveness has been linked to dopaminergic signaling and reward anticipation, and where individual differences in expectation may partially explain heterogeneity in treatment response.

Outcome selection and assessment strategy are also critical. The greater placebo sensitivity observed for UPDRS Part III compared with UPDRS Total suggests that domain-specific motor outcomes may be more vulnerable to contextual influences than composite measures of global disease severity. Several PD trials have relied primarily on UPDRS Part III assessed immediately after intervention [59,60,61], time point that may preferentially capture short-term placebo-related changes [15]. Future trials may benefit from prespecifying endpoint hierarchies that include both motor and global outcomes, while placing greater emphasis on follow-up assessments to evaluate durability. Complementing clinician-rated scales with instrumented or quantitative motor measures, such as wearable-derived gait metrics, tremor amplitude, or bradykinesia indices, may further reduce susceptibility to expectancy-related effects and improve objectivity [62,63,64,65].

Blinding procedures warrant particular attention in neuromodulation studies. Differences in sensory experience between active and sham stimulation—such as scalp sensation, sound, or muscle twitching—can compromise blinding and inadvertently enhance expectancy. Trials employing more sophisticated sham approaches, including specially designed sham coils or ramp-up/ramp-down stimulation in tDCS, have reported improved blinding credibility compared with earlier methods. Systematic assessment of blinding success, using participant and rater guesses of treatment allocation and confidence ratings, should be more consistently reported, as inadequate blinding may disproportionately influence placebo-sensitive outcomes [66,67].

The inclusion of mechanistic and biological outcome measures may further strengthen trial interpretation, although their limitations should be acknowledged. Neurophysiological measures (e.g., TMS-derived cortical excitability indices), neuroimaging markers (e.g., functional connectivity or task-related activation), and circulating biomarkers have been incorporated into some PD neuromodulation trials to support biological plausibility [45,68,69,70]. While these measures are not yet validated as surrogate clinical endpoints, they may be less susceptible to expectancy-related bias and can help distinguish transient placebo effects from sustained neuroplastic changes. Framing such measures as complementary secondary rather than definitive endpoints remain important.

Finally, alternative trial designs may help disentangle placebo and active effects more clearly. While two-arm active-versus-sham designs are standard, they inherently embed placebo effects in both groups. Importantly, the mere possibility of receiving placebo may attenuate observed benefits in the active arm—a phenomenon termed the “lessebo effect.” In a meta-analysis of Parkinson’s disease trials, Mestre et al. reported that active treatments in placebo-controlled studies produced 1.6-point smaller improvements in UPDRS motor scores (95% CI 0.2–3.0) compared with the same treatments evaluated in active-controlled trials, with larger differences observed in early disease and shorter studies [71]. This negative expectancy effect further complicates interpretation of two-arm active versus sham designs, as the comparison may underestimate true treatment efficacy. Where feasible, three-arm designs incorporating active stimulation, sham stimulation, and usual-care or no-intervention controls may provide a more direct estimate of placebo magnitude. Future studies using three-arm designs could help quantify both positive (placebo) and negative (lessebo) expectancy effects and provide more accurate partitioning of variance into placebo-related, active treatment-specific, and natural history components. For instance, open-label placebo demonstrated positive effects in many medical conditions such as depression and pain, as demonstrated in a meta-analysis [72]. Although such designs increase complexity and cost, they have been informative in other neuromodulation and behavioral intervention trials and may be particularly valuable in conditions like PD, where placebo responsiveness is well documented.

Collectively, these considerations suggest that future neuromodulation trials in Parkinson’s disease should place greater emphasis on expectancy management and measurement, outcome objectivity, assessment timing, and rigorous blinding. By integrating these design features, studies may reduce heterogeneity, improve the separation of placebo and active effects, and enhance the interpretability and translational relevance of neuromodulation research in PD.

### 4.3. Strengths and Limitations

This review and meta-analysis aimed to evaluate placebo effects across the available data on randomized controlled trials. A key strength is the examination of both within-group and between-group effects across post-intervention and follow-up time points, allowing assessment of the temporal dynamics of placebo and active responses, as well as the comparison of composite and domain-specific outcomes. Important limitations to acknowledge include the modest number of eligible studies as well as the heterogeneity in stimulation parameters and participant characteristics. Although the search strategy encompassed several non-invasive neuromodulation modalities, only rTMS and tDCS trials met the eligibility criteria, reflecting the current state of the available evidence. Incomplete reporting of raw data limited the ability to conduct more diverse analyses [40,41,42,43], the reason why publication bias cannot be excluded. The heterogeneity observed in our analyses is likely related to differences in study and participant characteristics, including stimulation frequency and intensity, number of sessions, disease severity, medication state (ON/OFF), and follow-up duration—parameters that represent common sources of variability in neuromodulation studies for Parkinson’s disease. Meta-regression or sensitivity analyses were not performed because the limited number of studies per comparison precluded reliable subgroup analyses. Moreover, although all included trials were sham-controlled, most did not include a third no-treatment or usual-care control arm, which limits the ability to fully separate placebo-related effects from the natural course of the disease and the specific effects of the active intervention. Accordingly, changes observed within sham groups should not be interpreted as pure placebo effects. In a fluctuating disorder such as Parkinson’s disease, these changes likely reflect a composite of placebo-related expectancy, natural disease variability, regression to the mean, and measurement error. Therefore, within-sham group results should be interpreted as this combined nonspecific signal rather than an isolated or causal placebo response. In addition, the available follow-up periods were predominantly short- to medium-term, which limits inferences about long-term placebo or active effects. Thus, future studies should adopt trial designs incorporating control groups alongside active and sham conditions, standardized reporting of stimulation parameters and outcomes, and longer follow-up periods to better distinguish short-term placebo-related effects from sustained neuromodulatory efficacy. Collectively, these approaches would enhance mechanistic interpretation, reduce bias, and strengthen the clinical translation of neuromodulation interventions in PD.

## 5. Conclusions

This study shows that placebo effects contribute to short-term clinical improvement in PD neuromodulation trials, particularly for motor outcomes, but do not fully explain the sustained benefits observed with active stimulation. Placebo-related improvements were most prominent immediately after intervention and attenuated over time, whereas active neuromodulation demonstrated more consistent and durable effects. The divergence between global and motor outcomes, as well as between immediate and follow-up assessments, highlights the outcome- and time-dependence of placebo responsiveness in PD. Early post-intervention assessments may therefore preferentially capture expectancy-driven effects, while follow-up evaluations provide a more reliable estimate of stimulation-specific therapeutic impact. Together, these findings emphasize the importance of rigorous trial design, including careful outcome selection, appropriate timing of assessments, and comparator structures that allow separation of placebo and active effects. Longer follow-up periods and, where feasible, the inclusion of control arms in addition to sham conditions will be critical for accurately quantifying true neuromodulatory efficacy and supporting clinical translation in PD.

## Data Availability

No new data were created or analyzed in this study.

## Supplementary Materials

The following supporting information can be downloaded at: https://www.mdpi.com/article/10.3390/biomedicines14030532/s1, Figure S1: Risk of bias of studies included; Table S1: Search strategies for the online databases PubMed (NCBI), and Embase, Cochrane. Table S2: Study Characteristics. AMT: active motor threshold, DLPFC: dorsolateral prefrontal cortex, iTBS: intermittent theta-burst stimulation, M1: primary motor cortex, PFC: prefrontal cortex, RCT: randomized clinical trial, RMT: resting motor threshold, rTMS: repetitive transcranial magnetic stimulation, SMA: supplementary motor area. File S1: PRISMA 2020 checklist.
