# Supplementary material for: Reconsidering Placebo Effects in Neuromodulation for Parkinson’s Disease: Lessons for Clinical Trials and Therapeutic Translation
Source: Biomedicines. 2026 Feb 27;14(3):532. doi: 10.3390/biomedicines14030532 (PMC13024062; doi:10.3390/biomedicines14030532)
Supplement: Supplementary file 1 [file biomedicines-14-00532-s001.zip › Supplementary Material.pdf]

Supplementary Material

Table S1. Search strategies for the online databases PubMed (NCBI), and Embase, Cochrane.

**PubMed (NCBI)**
("Parkinson Disease"[Mesh] OR Parkinson[tiab] OR "Parkinson's disease"[tiab] OR "Parkinson disease"[tiab] OR PD[tiab]) AND ("Placebos"[Mesh] OR "Placebo Effect"[Mesh] OR placebo[tiab] OR placebos[tiab] OR "placebo-controlled"[tiab] OR sham[tiab] OR "sham stimulation"[tiab] OR "sham control"[tiab] OR "sham group"[tiab]) AND ("Unified Parkinson's Disease Rating Scale"[tiab] OR "Unified Parkinson Disease Rating Scale"[tiab] OR UPDRS[tiab]) AND ("Transcranial Magnetic Stimulation"[Mesh] OR "Transcranial Direct Current Stimulation"[Mesh] OR "Vagus Nerve Stimulation"[Mesh] OR "Electric Stimulation Therapy"[Mesh] OR "Magnetics"[Mesh] OR "non-invasive brain stimulation"[tiab] OR "repetitive transcranial magnetic stimulation"[tiab] OR rTMS[tiab] OR TMS[tiab] OR "theta burst stimulation"[tiab] OR TBS[tiab] OR "transcranial direct current stimulation"[tiab] OR tDCS[tiab] OR "vagus nerve stimulation"[tiab] OR "vagal nerve stimulation"[tiab] OR VNS[tiab] OR tVNS[tiab] OR nVNS[tiab] OR "static magnetic field stimulation"[tiab] OR SMS[tiab] OR tSMS[tiab] OR "transcranial pulse stimulation"[tiab] OR TPS[tiab] OR "transcranial pulsed ultrasound"[tiab] OR TPU[tiab]) AND ("clinical trial"[Publication Type] OR "randomized controlled trial"[Publication Type])
**Embase**
('parkinson disease'/exp OR 'parkinson disease' OR parkinson:ti,ab OR parkinsons:ti,ab OR 'parkinson disease':ti,ab OR pd:ti,ab) AND ('placebo'/exp OR placebo OR 'placebo effect'/exp OR 'placebo effect' OR placebo:ti,ab OR placebos:ti,ab OR 'placebo controlled':ti,ab OR sham:ti,ab OR 'sham control':ti,ab OR 'sham stimulation':ti,ab OR 'sham group':ti,ab) AND (updrs:ti,ab OR 'unified parkinson disease rating scale':ti,ab OR 'unified parkinsons disease rating scale':ti,ab) AND ('transcranial magnetic stimulation'/exp OR 'transcranial magnetic stimulation' OR 'transcranial direct current stimulation'/exp OR 'transcranial direct current stimulation' OR 'vagus nerve stimulation'/exp OR 'vagus nerve stimulation' OR 'electric stimulation therapy'/exp OR 'electric stimulation therapy' OR 'magnetics'/exp OR magnetics OR rtms:ti,ab OR tms:ti,ab OR tdcs:ti,ab OR 'theta burst stimulation':ti,ab OR tbs:ti,ab OR 'vagus nerve stimulation':ti,ab OR 'vagal nerve stimulation':ti,ab OR vns:ti,ab OR tvns:ti,ab OR nvns:ti,ab OR 'static magnetic field stimulation':ti,ab OR sms:ti,ab OR tsms:ti,ab OR 'transcranial pulse stimulation':ti,ab OR tps:ti,ab OR 'transcranial pulsed ultrasound':ti,ab OR tpu:ti,ab OR 'non-invasive brain stimulation':ti,ab) AND ('randomized controlled trial'/exp OR 'clinical trial'/exp OR randomized controlled trial:ti,ab OR clinical trial:ti,ab)
**Cochrane - CENTRAL**
([mh "Parkinson Disease"] OR Parkinson:ti,ab,kw OR "Parkinson disease":ti,ab,kw OR "Parkinson's disease":ti,ab,kw OR PD:ti,ab,kw) AND ([mh "Placebos"] OR [mh "Placebo Effect"] OR placebo:ti,ab,kw OR placebos:ti,ab,kw OR placebo-controlled:ti,ab,kw OR sham:ti,ab,kw OR "sham stimulation":ti,ab,kw OR "sham control":ti,ab,kw OR "sham group":ti,ab,kw) AND ("Unified Parkinson Disease Rating Scale":ti,ab,kw OR "Unified Parkinson's Disease Rating Scale":ti,ab,kw OR UPDRS:ti,ab,kw) AND ([mh "Transcranial Magnetic Stimulation"] OR [mh "Transcranial Direct Current Stimulation"] OR [mh "Vagus Nerve Stimulation"] OR [mh "Electric Stimulation Therapy"] OR [mh "Magnetics"] OR "non-invasive brain stimulation":ti,ab,kw OR "repetitive transcranial magnetic stimulation":ti,ab,kw OR rTMS:ti,ab,kw OR TMS:ti,ab,kw OR "theta burst stimulation":ti,ab,kw OR TBS:ti,ab,kw OR "transcranial direct current stimulation":ti,ab,kw OR tDCS:ti,ab,kw OR "vagus nerve stimulation":ti,ab,kw OR "vagal nerve stimulation":ti,ab,kw OR VNS:ti,ab,kw OR tVNS:ti,ab,kw OR nVNS:ti,ab,kw OR "static magnetic field stimulation":ti,ab,kw OR SMS:ti,ab,kw OR tSMS:ti,ab,kw OR "transcranial pulse stimulation":ti,ab,kw OR TPS:ti,ab,kw OR "transcranial pulsed ultrasound":ti,ab,kw OR TPU:ti,ab,kw)

Table S2. Study Characteristics. AMT: active motor threshold, DLPFC: dorsolateral prefrontal cortex, iTBS: intermittent theta-burst stimulation, M1: primary motor cortex, PFC: prefrontal cortex, RCT: randomized clinical trial, RMT: resting motor threshold, rTMS: repetitive transcranial magnetic stimulation, SMA: supplementary motor area.

Study	Study Design	Total Sample Size	Placebo Group	Active Group	Age (year)	Sex	Time Since PD Diagnosis (year)	Type of Intervention	Number of Sessions	Session Duration (min)	Stimulation Site	Intensity	Frequency	Number of Pulses	Sham Intervention Protocol	First Follow-Up Time Point (days)	Medication Status During the Assessments
Shirota 2013	RCT	106	36	70	67.5 ± 8.2	45 M, 61 F	8.0 ± 6.2	rTMS	8	17–20	SMA	110% AMT	1 Hz / 10 Hz	1000	Electrical skin stimulation synchronized with sham TMS	84	ON
Aftanas 2022	RCT	47	25	22	63.7 ± 2.3	22 M, 25 F	6.3 ± 1.5	rTMS	20	40	Bilateral M1 and left DLPFC	100% RMT (M1); 110% RMT (DLPFC)	10 Hz	4000 (M1); 3000 (DLPFC)	Placebo coil	-	-
Benninger 2011	RCT	26	13	13	63.9 ± 8.1	18 M, 8 F	8.7 ± 5.9	iTBS	8	-	Bilateral M1 and	80% AMT	50 Hz	600/site	Sham coil	28	OFF

											DLPFC						
Benninger 2012	RCT	26	13	13	64.1 ± 8.5	20 M, 6 F	9.0 ± 5.5	rTMS	8	-	Bilateral M1	80% AMT	50 Hz	300/site	Inactive coil	28	OFF
Börnke 2004	Cross-over	12	12	12	55.9 ± 13.1	6 M, 6 F	-	rTMS	1	-	Motor hand area	90% RMT	10 Hz	1000	Angled coil sham	0,14	OFF
Mi 2019	RCT	30	10	20	63.6 ± 9.9	14 M, 16 F	8.6 ± 5.5	rTMS	10	20	SMA	90% RMT	10 Hz	1000	Coil angled 90°	14	ON
Okabe 2003	RCT	85	28	57	67.2 ± 8.2	48 M, 37 F	8.5 ± 5.6	rTMS	8	-	Motor cortex / occipital	110% AMT	0.2 Hz	100	Electrical sham	56	Moderate
Aftanas 2021	RCT	46	23	23	63.3 ± 7.9	21 M, 25 F	6.3 ± 4.0	rTMS	20	40	Bilateral M1 and left DLPFC	100–110% RMT	10 Hz	4000 (M1); 3000 (DLPFC)	Placebo coil	28	-
Zhuang 2020	RCT	33	14	19	61.0 ± 10.9	18 M, 15 F	5.8 ± 4.1	rTMS	10	20	Right DLPFC	110% RMT	1 Hz	1200	Flipped coil sham	28	OFF
Shaheen 2023	RCT	40	20	20	61.4 ± 6.7	22 M, 18 F	3.5 ± 2.2	rTMS	12	-	Bilateral parietal	100% RMT	10 Hz	1000	Tilted coil sham	28	-

Spagnolo 2021	RCT	60	20	39	67.5 ± 8.0	41 M, 18 F	6.9 ± 3.5	rTMS	12	-	M1 and PFC	90–100% RMT	10 Hz	840/site	Sham H-coil	28	ON/OFF
Valentino 2014	Cross-over	10	10	10	72.3 ± 3.6	5 M, 5 F	11.0 ± 4.9	tDCS	5	20	M1	2 mA	DC	-	Sham 30 sec	2	ON
Benninger 2010	RCT	25	12	13	63.9 ± 8.7	16 M, 9 F	9.9 ± 5.6	tDCS	8	20	Motor cortex and PFC	2 mA	DC	-	Short DC sham	28	OFF
da Silva 2018	RCT	21	10	11	66.0 ± 7.8	10 M, 7 F	5.5 ± 4.1	tDCS	1	15	M1 and SMA	2 mA	DC	-	30 sec sham	-	ON
Fregni 2006	Cross-over	17	9	9	62.3 ± 1.6	11 M, 6 F	12.3 ± 1.6	tDCS	1	20	Left M1 / DLPFC	1 mA	DC	-	Current off after 5 sec	-	OFF
Ji 2021	RCT	42	20	22	61 ± 8.1	28 M, 14 F	4.8 ± 3.1	cTBS	14	-	Left SMA	80% RMT	5 Hz	600	Mimicked sound and sensation	15	OFF
Ma 2019	RCT	28	10	8	62.1 ± 9.39	13 M, 15 F	8.43 ± 5.21	rTMS	10	20	SMA	90% RMT	10 Hz	1000	Coil angled 90° over SMA	15	OFF

AMT, active motor threshold; DC, direct current; DLPFC, dorsolateral prefrontal cortex; iTBS, intermittent theta-burst stimulation; cTBS, theta-burst stimulation; M1, primary motor cortex; PD, Parkinson's disease; PFC, prefrontal cortex; RCT, randomized clinical trial; RMT, resting motor threshold; rTMS, repetitive transcranial magnetic stimulation; SMA, supplementary motor area; tDCS, transcranial direct current stimulation; TMS, transcranial magnetic stimulation; Hz, hertz; mA, milliampere; min, minutes.

Figure S1. Risk of bias of studies included.

Study ID	D1	D2	D3	D4	D5	Overall
Shirota 2013	+	+	!	+	!	!
Aftanas 2022	!	+	!	+	!	!
Benninger 2011	!	+	!	!	!	!
Benninger 2012	!	+	!	+	!	!
Börnke 2004	!	!	+	!	!	!
Mi 2019	+	+	+	+	!	!
Okabe 2003	!	+	!	+	!	!
Aftanas 2021	!	+	!	+	!	!
Zhuang 2020	+	+	+	+	!	!
Shaheen 2023	!	+	!	!	!	!
Spagnolo 2021	+	+	+	+	!	!
Valentino 2014	!	!	+	!	!	!
Benninger 2010	!	+	!	+	!	!
da Silva 2018	!	+	!	!	!	!
Fregni 2006	!	!	+	!	!	!

Ma 2019	!	+	!	+	!	!
Ji 2021	!	+	+	+	!	!

D1, bias arising from the randomization process; D2, bias due to deviations from intended interventions; D3, bias due to missing outcome data; D4, bias in measurement of the outcome; D5, bias in selection of the reported result. Risk-of-bias judgments were assigned according to the Cochrane Risk of Bias (RoB 2) tool, where "+" indicates low risk of bias, "!" indicates some concerns, and "–" indicates high risk of bias.
